# IgG Abnormality in Narcolepsy and Idiopathic Hypersomnia

**DOI:** 10.1371/journal.pone.0009555

**Published:** 2010-03-05

**Authors:** Susumu Tanaka, Makoto Honda

**Affiliations:** Research on the Cause and Treatment of Sleep Disorders, Tokyo Institute of Psychiatry, Tokyo, Japan; Emory University, United States of America

## Abstract

**Background:**

A close association between narcolepsy and the Human Leukocyte Antigen (HLA)-DQB1*0602 allele suggests the involvement of the immune system, or possibly an autoimmune process. We investigated serum IgG levels in narcolepsy.

**Methodology/Principal Findings:**

We measured the serum total IgG levels in 159 Japanese narcolepsy-cataplexy patients positive for the HLA-DQB1*0602 allele, 28 idiopathic hypersomnia patients with long sleep time, and 123 healthy controls (the HLA-DQB1*0602 allele present in 45 subjects). The serum levels of each IgG subclass were subsequently measured. The distribution of serum IgG was significantly different among healthy controls negative for the HLA-DQB1*0602 allele (11.66±3.55 mg/ml), healthy controls positive for the HLA-DQB1*0602 allele (11.45±3.43), narcolepsy patients (9.67±3.38), and idiopathic hypersomnia patients (13.81±3.80). None of the following clinical variables, age, disease duration, Epworth Sleepiness Scale, smoking habit and BMI at the time of blood sampling, were associated with IgG levels in narcolepsy or idiopathic hypersomnia. Furthermore we found the decrease in IgG1 and IgG2 levels, stable expression of IgG3, and the increase in the proportion of IgG4 in narcolepsy patients with abnormally low IgG levels. The increase in the proportion of IgG4 levels was also found in narcolepsy patients with normal serum total IgG levels. Idiopathic hypersomnia patients showed a different pattern of IgG subclass distribution with high IgG3 and IgG4 level, low IgG2 level, and IgG1/IgG2 imbalance.

**Conclusions/Significance:**

Our study is the first to determine IgG abnormalities in narcolepsy and idiopathic hypersomnia by measuring the serum IgG levels in a large number of hypersomnia patients. The observed IgG abnormalities indicate humoral immune alterations in narcolepsy and idiopathic hypersomnia. Different IgG profiles suggest immunological differences between narcolepsy and idiopathic hypersomnia.

## Introduction

Narcolepsy is a chronic sleep disorder characterized by excessive daytime sleepiness and cataplexy [1]. Narcolepsy bears a remarkable association with Human Leukocyte Antigen (HLA)-DQB1*0602 allele, suggesting that an autoimmune process underlies the pathophysiology of narcolepsy [2]. Several studies based on the autoimmune hypothesis have been conducted on possible narcolepsy-related antigens or the screening of novel autoantibodies against the hypothalamus [3], [4], [5], [6], [7], [8], [9], [10]. Thus far, however, no studies have furnished notable results for obtaining autoantibodies related to narcolepsy.

Recently, Jackson et al. showed that immunoglobulin G (IgG), which was purified and concentrated from narcolepsy patients, bound to membrane proteins and modified colon function [11]. Potential reasons why conventional methods fail to detect such functional autoantibodies are the denaturation of target antigens, extremely low concentrations of high affinity antibody, and low levels of initial total IgG level in the serum of narcolepsy patients. Our previous studies for autoantibody screening showed that the autoantibody indices of narcolepsy patients were generally low and sometimes below the mean minus 2SD of healthy controls [5], [10]. This finding is in line with the last possible reason for the failure in autoantibody detection. Furthermore, a case of common variable immunodeficiency syndrome (IgG2 and IgG4 deficiency) has been reported after the onset of narcolepsy [12]. Thus, high-dose intravenous immunoglobulins (IVIg), which have been shown to decrease the frequency and severity of cataplexy in some narcolepsy patients [13], might be effective not only through the assumed neutralization by anti-idiotypic autoantibody but through supplementation of low IgG. In the present study, we measured the serum levels of total IgG and four IgG subclasses in narcolepsy-cataplexy patients and compared these values with those in healthy controls and in patients with typical idiopathic hypersomnia with long sleep time.

## Materials and Methods

This research was approved by the ethics committees of the Neuropsychiatric Research Institute (Tokyo, Japan) and the Tokyo institute of Psychiatry Ethics Committee. Written informed consents were obtained from all participants. All the patients were clinically diagnosed at the Neuropsychiatric Research Institute according to the diagnostic criteria defined in the International Classification of Sleep Disorders, second edition [1]. Five mL of blood was drawn and the sera were stored at −80C until use at the Tokyo Institute of Psychiatry or at the Neuropsychiatric Research Institute. Clinical data relating to sleep habits were obtained and used for analysis. Healthy subjects were excluded from the study if they reported excessive daytime sleepiness or any signs of immunological abnormalities in the questionnaire collected at the time of blood collection.

Patient demographic data are summarized in Table 1. All the narcolepsy patients had definite cataplexy and were positive for HLA-DQB1*0602 allele. Sera from 159 narcolepsy-cataplexy patients, 28 patients with idiopathic hypersomnia with long sleep time, and 123 healthy controls (78 negative and 45 positive for HLA-DQB1*0602 allele; they were hereinafter referred to as HLA-negative controls and HLA-positive controls, respectively) were examined. HLA typing for HLA-DRB1 and DQB1 loci were performed for all subjects at the NPO HLA Laboratory (Kyoto, Japan) or by the Wakunaga Pharmaceutical Company (Hiroshima, Japan). We analyzed potential relationships of the following clinical data with the IgG level: age, sex, disease duration, body mass index (BMI), smoking habits and Epworth Sleepiness Scale (ESS) obtained at the time of blood collection, and past history of autoimmune disorders. Since smoking is shown to be associated with decreased level of IgG [14], we analyzed the effect of smoking habits in details.

**Table 1:** Profile of the subjects.

	n	Age (years, mean ± SD)	Sex, no. male/no. female	IgG concentration (mg/ml)			
				range	mean ± SD	below normal range	above normal range
Healthy controls	123	41.18±11.69	69/54	5.59–22.26	11.58±3.49	0	1
HLA-DQB1*0602 negative	78	42.19±12.22	49/29	5.59–22.26	11.66±3.55	0	1
HLA-DQB1*0602 positive	45	39.35±10.56	20/25	7.23–21.15	11.45±3.43	0	0
Narcolepsy with cataplexy	159	46.63±17.30	96/63	3.26–24.36	9.67±3.38*	12	1
Idiopathic hypersomnia	28	27.18±10.95*	11/17	7.75–22.80	13.81±3.80*	0	3

The Mann– Whitney U-test was used to compare IgG concentration among patient groups and healthy controls.

*The mean values were significantly different from healthy controls negative for HLA-DQB1*0602 allele at p<0.05.

To examine the details of total IgG abnormality especially in a subgroup of narcolepsy patients with abnormally low IgG level, we subsequently selected 67 samples from 245 initial experiments and measured the IgG subclass in 23 narcolepsy patients (total IgG levels normal in 13 patients and abnormally low in 10), 14 HLA-negative controls, 13 HLA-positive controls, and 17 idiopathic hypersomnia patients.

The serum IgG levels were measured using the Easy-Titer Human IgG (H+L) Assay Kit (Pierce Biotechnology Inc., IL) based on monodispersed polystyrene beads. The beads are coated with anti-human IgG and absorb light at 340 and 405 nm. Once the anti-human IgG on the beads bind to serum IgG, the beads aggregate and cause a decrease in the light absorption. The normal range of the serum total IgG measured by this kit is 5.5–22.0 mg/ml according to the manufacturer's instructions. The serum total IgG levels below or above this range are considered to be abnormal.

Human IgG molecules are subdivided into four classes: IgG1–4. The low level of each subclass or a combination of these subclasses is found in congenital or secondary immunodeficiency diseases [15]. Therefore, we next measured the serum levels of four IgG subclasses to examine the characteristics of IgG subclass profiles especially in narcolepsy patients with abnormally low total IgG level. These serum levels were compared among 10 narcolepsy patients with low serum IgG levels, 13 age-matched narcolepsy patients with normal serum IgG levels, 13 age-matched HLA-negative controls, 14 age-matched HLA-positive controls (all the controls showed normal total IgG level), and 17 idiopathic hypersomnia patients (two of which were positive for HLA-DQB1*0602 allele). The serum level of each IgG subclass was determined using the human IgG subclass profile ELISA kit (Invitrogen, Carlsbad, CA) based on a sandwich-type ELISA method.

The proportion of each IgG subclass in each subject was calculated and expressed as a ratio of the value to the total of IgG1, IgG2, IgG3, and IgG4. Since the IgG subclasses were not normally distributed, we followed the recommendation for setting normal ranges [16] and determined the normal range of each IgG subclass as the mean ± 2SD in the 13 HLA-negative controls.

Distribution of the total IgG and IgG subclass levels among groups of narcolepsy, idiopathic hypersomnia, and healthy controls was compared using ANOVA and the Mann-Whitney *U* test. Significance levels are set at p<0.0125 for total IgG and p<0.01 for each IgG subclass after Bonferroni correction for multiple comparisons. We assessed the effect of age, disease duration, ESS, and BMI on total IgG levels using multivariate regression analysis. We also compared the distribution of serum total IgG levels according to sex difference and smoking habit by using the Mann-Whitney *U* test.

## Results

**Figure 1:**
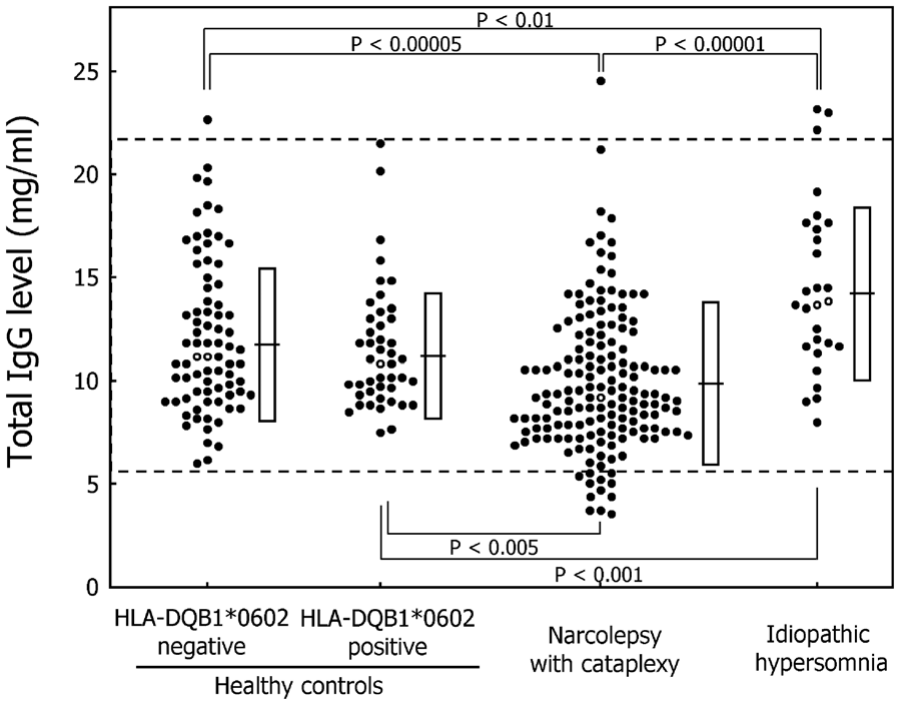
Serum total IgG level. Each dot corresponds to the serum IgG level (mg/ml) in each subject. Dotted lines indicate the normal range. The normal range is set at 5.5–22.0 mg/ml according to the manufacturer's instructions. Horizontal lines and squares indicate the mean ± SD of four groups. Outlined-dots indicate the median of four groups.

The mean total IgG level showed significant differences among groups: it was lower in narcolepsy-cataplexy patients (mean ± SD: 9.67±3.38, range: 3.26–24.36 mg/ml, p<0.00005) and higher in idiopathic hypersomnia patients (13.81±3.80, 7.75–22.80 mg/ml, p<0.01) than in HLA-negative controls (11.66±3.55, 5.59–22.26 mg/ml) (Fig. 1, Table 1). The mean total IgG level was also lower in narcolepsy-cataplexy patients (p<0.005) and higher in idiopathic hypersomnia patients with long sleep time (p<0.001) than in HLA-positive controls (11.45±3.43, 7.23–21.15 mg/ml).

The results of Thompson's rejection test revealed that two samples from HLA-positive controls with high IgG levels were outliers as compared to the remaining 43 HLA-positive controls. After omitting these two outliers, we still observed significant differences in the mean total IgG level between narcolepsy patients and HLA-positive controls (p<0.005). One healthy control, one narcolepsy patient, and three idiopathic hypersomnia patients showed abnormally high serum total IgG levels above the normal range. Instead, 12 male narcolepsy patients showed abnormally low total IgG levels.

None of the following clinical variables, age, disease duration, ESS score, smoking habit, and BMI at the time of blood sampling, were independently associated with IgG levels in narcolepsy patients and idiopathic hypersomnia patients. The serum total IgG level in smokers was significantly lower than that in non-smoker among healthy controls (p<0.05), confirming the previous report [14] (HLA-negative controls without smoking: 12.82±3.61, HLA-negative controls with smoking; 10.61±2.41, HLA-positive controls without smoking: 11.72±3.18, HLA-positive controls with smoking; 9.67±2.23) (Figure S1). Epidemiological study suggests that narcolepsy patients had increased risk of passive smoking [17]. However, the decrease in the serum total IgG level in narcolepsy patients was observed regardless of smoking habits, and there were no significant difference between smoking group and non-smoking group in narcolepsy patients (9.22±2.80 and 9.95±3.21, respectively). Idiopathic hypersomnia patients (all non-smokers) showed marginally high total IgG level (14.97±4.07) when compared with non-smoking controls (p = 0.053). Data regarding the smoking habit were available in 4 of 12 narcolepsy patients with abnormally low total IgG level; one was non-smoker and the others were smokers. In non-smoking controls, we found a sex difference in total IgG level according to the HLA-status; the mean total IgG level of HLA-positive male controls was marginally low (10.48±2.21) compared to those of HLA-negative male controls (13.09±3.56) (p = 0.031), while no significant difference was found between HLA-positive (12.30±3.45) and negative (12.25±3.81) female controls (p = 0.451). We also analyzed the effect of psycho-stimulant medication on the low total IgG level. Four of the 12 narcolepsy patients with low IgG levels were un-medicated at the time of blood sampling. No differences were observed in the serum total IgG levels between 8 medicated and 4 un-medicated narcolepsy patients with low IgG levels.

We next analyzed the serum levels of four IgG subclasses in 23 narcolepsy patients (total IgG levels were low in 10 patients and within the normal range in 13), 27 healthy controls (13 HLA-negative and 14 HLA-positive) and 17 idiopathic hypersomnia patients. The results of mean IgG1, IgG2, IgG3, and IgG4 levels, the IgG1/IgG2 ratio, and the estimated normal range determined from 13 HLA-negative controls are summarized in Table 2. The mean percentage of each IgG subclass in the summation of the four IgG subclasses and the calculated reference ranges are summarized in Table 3.

**Table 2:** Four IgG subclasses (mg/ml).

	n	IgG1	IgG2	IgG1/IgG2 ratio	IgG3	IgG4
Healthy controls						
HLA-DQB1*0602 negative	13	6.76±1.23	4.07±1.30	1.80±0.58	0.30±0.11‡	0.49±0.19
HLA-DQB1*0602 positive	14	6.23±1.27	3.13±1.12	2.31±1.24	0.33±0.10‡	0.41±0.31♯
Narcolepsy						
with normal range of IgG	13	6.15±1.06	3.73±1.56	1.96±0.90	0.40±0.14	0.82±0.50
with low IgG levels	10	4.65±2.42*	2.71±1.24†	2.40±2.11	0.28±0.16‡	0.59±0.63
Idiopathic hypersomnias	17	7.39±2.81	2.95±1.11†	2.67±0.93†	0.55±0.34†	0.92±1.12
Normal range estimated		4.30–9.22	1.46–6.68	0.65–2.96	0.07–0.55	0.11–0.88

*P<0.01 amongst healthy controls negative, positive for HLA-DQB1*0602 allele, narcolepsy with normal range of IgG and idiopathic hypersomnias.

† The Value showed a raising/declining tendency against healthy controls negative for HLA-DQB1*0602 allele (P<0.03).

‡ P<0.03 against idiopathic hypersomnias.

♯ P<0.03 against narcolepsy patients with normal range of IgG.

**Table 3:** The proportion of each IgG subclass in total IgG (%).

	n	IgG1	IgG2	IgG3	IgG4
Healthy controls					
HLA-DQB1*0602 negative	13	58.55±5.79	34.48±6.68	2.68±1.05	4.30±1.41
HLA-DQB1*0602 positive	14	61.87±9.54	30.70±8.53	3.38±1.31	4.06±2.86
Narcolepsy					
with normal range of IgG	13	56.77±9.79	32.55±9.10	3.67±1.23*	7.06±3.92
with low IgG levels	10	56.60±16.71	33.29±13.95	3.35±1.61	6.83±6.96
Idiopathic hypersomnias	17	62.85±5.61	25.66±6.77**	4.79±2.93**	6.70±4.70
Normal range estimated		46.97–70.14	21.12–47.84	0.57–4.78	1.47–7.12

*P<0.03,

**P<0.01 against healthy controls negative for HLA-DQB1*0602 allele.

**Figure 2:**
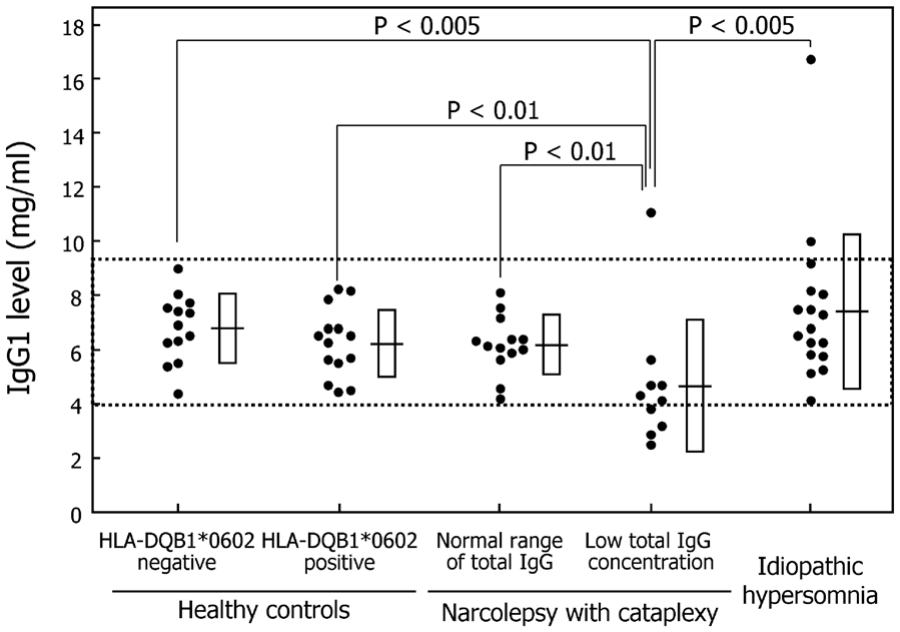
Serum IgG1 level. Each dot corresponds to the serum IgG subclass level (mg/ml) in each subject. Dotted lines indicate the mean ± 2SD of healthy control subjects without DQB1*0602 as normal range. Horizontal lines and squares indicate the mean ± SD of five groups.

The distribution of each IgG subclass showed significant or marginally significant differences among 5 groups by analysis of variance, therefore we studied the details to clarify the characteristics of narcolepsy patients with low total IgG levels and idiopathic hypersomnia patients with long sleep time as follows;

IgG1: The mean IgG1 levels were significantly lower in narcolepsy patients with low total IgG levels than in HLA-negative controls (p<0.005), HLA-positive controls (p<0.01), narcolepsy patients with normal total IgG levels (p<0.01), and idiopathic hypersomnia patients (p<0.005) (Table 2, Fig. 2). Five narcolepsy patients with low total IgG levels and two idiopathic hypersomnia patients had abnormal IgG1 levels (Fig. 2).

**Figure 3:**
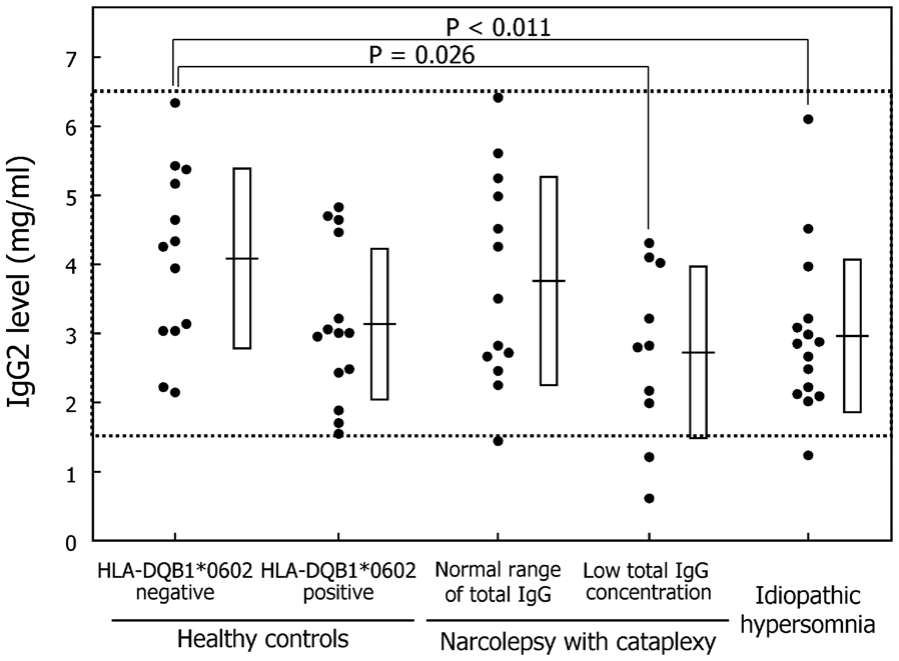
Serum IgG2 level. The definitions of dots, dotted lines, horizontal lines, and squares are provided in the legend for Figure 2.

IgG2: The mean IgG2 levels in idiopathic hypersomnia patients (p = 0.011) tended to decrease, and those in the subgroup of narcolepsy patients with low total IgG levels tended to decrease (p = 0.026) compared with the mean IgG2 levels in HLA-negative controls (Table 2, Fig. 3). Three narcolepsy patients and one idiopathic hypersomnia patient presented low IgG2 levels below the normal range. The mean proportion of IgG2 in idiopathic hypersomnia patients was significantly lower than that in HLA-negative controls (Table 3).

**Figure 4:**
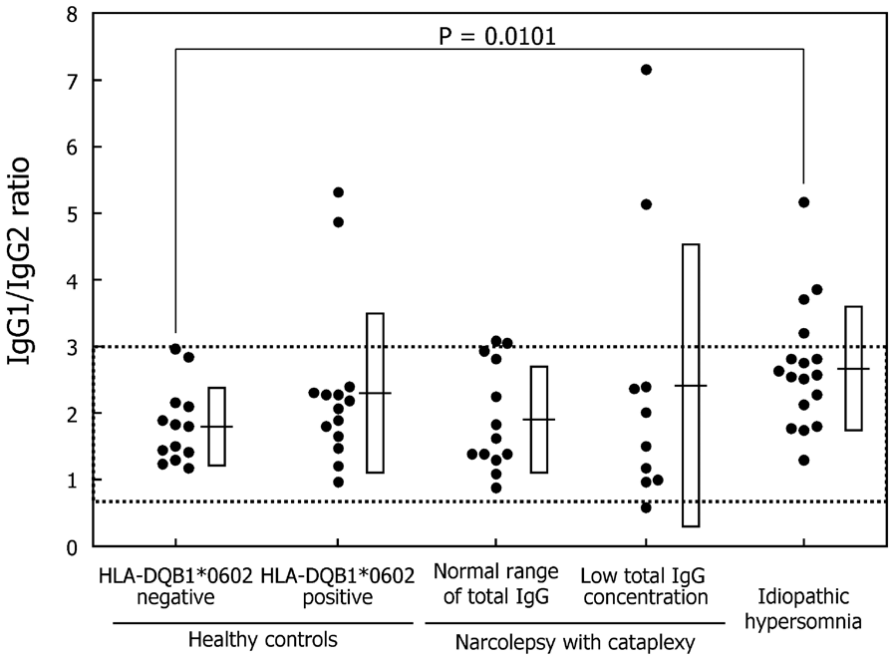
IgG1/IgG2 ratio. Dots correspond to the IgG1/IgG2 ratio in each subject. The definitions of dotted lines, horizontal lines, and squares are provided in the legend for Figure 2.

IgG1/IgG2 ratio: The mean IgG1/IgG2 ratios tended to increase in idiopathic hypersomnia patients as compared with those in HLA-negative controls (p = 0.0101) (Table 2, Fig. 4). Two HLA-positive controls, five narcolepsy patients, and four idiopathic hypersomnia patients showed abnormal IgG1/IgG2 ratios.

**Figure 5:**
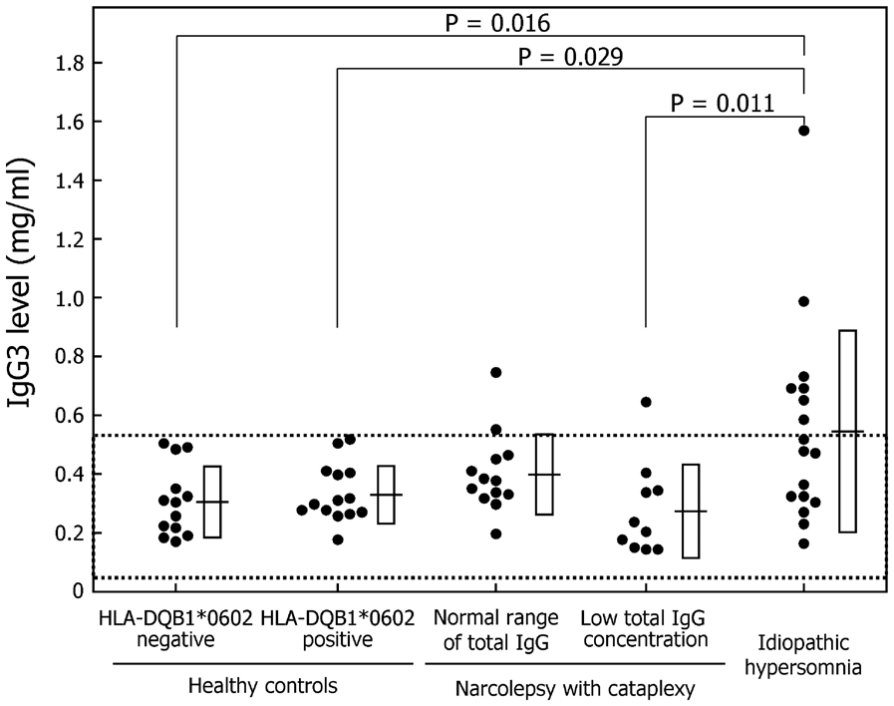
Serum IgG3 level. Definitions of dots, dotted lines, horizontal lines, and squares are provided in the legend for Figure 2.

IgG3: The mean IgG3 levels tend to be higher in idiopathic hypersomnia patients than in HLA-negative controls (p = 0.016), HLA-positive controls (p = 0.029), and narcolepsy patients with low IgG level (p = 0.0105) (Table 2, Fig. 5). Abnormally high IgG3 levels were found in three narcolepsy patients and seven idiopathic hypersomnia patients. The mean proportion of IgG3 significantly increased in idiopathic hypersomnia patients (p<0.01) and tended to be higher in the narcolepsy patients with normal IgG levels (p = 0.026) than in HLA-negative controls (Table 3).

**Figure 6:**
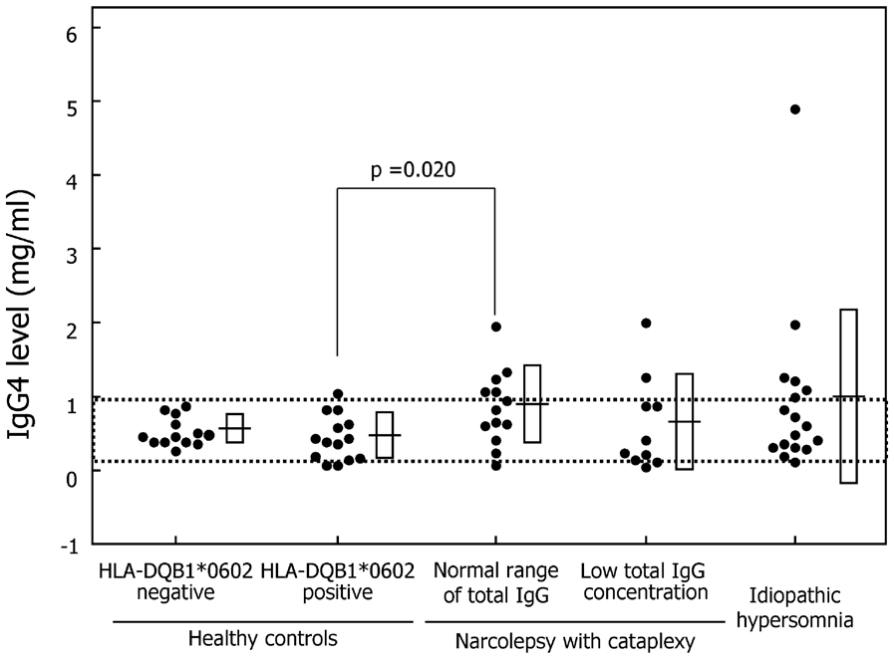
Serum IgG4 level. Definitions of dots, dotted lines, horizontal lines, and squares are provided in the legend for Figure 2.

IgG4: Narcolepsy patients with normal IgG levels tended to have higher IgG4 levels as compared to HLA-positive controls (p = 0.020) (Table 2, Fig. 6). The mean IgG4 levels were relatively higher in narcolepsy patients and idiopathic hypersomnia patients, but these differences were not statistically significant (Table 2). Abnormal IgG4 levels were found in one HLA-positive control, 10 narcolepsy patients, and 7 idiopathic hypersomnia patients. The mean proportion of IgG4 significantly increased in both narcolepsy patients and idiopathic hypersomnia patients regardless of total IgG levels as compared with that in healthy controls (Table 3)

## Discussion

Our study is the first to determine IgG abnormalities in narcolepsy and idiopathic hypersomnia by measuring the serum IgG levels in a large number of hypersomnia patients. Decrease in the serum total IgG levels and stable IgG3 level were found in a subgroup of narcolepsy patients with abnormally low total IgG levels. The elevated proportion of IgG4 subclass was a common finding in narcolepsy patients regardless of serum total IgG levels. On the other hand, elevated total IgG levels, IgG3, IgG4, and IgG1/IgG2 ratio and low IgG2 level were found in idiopathic hypersomnia patients. Our data indicated a significant increase in the IgG1/IgG2 ratio in idiopathic hypersomnia patients, suggesting a Th1 involvement in idiopathic hypersomnia. Different IgG profiles between these two hypersomnias indicate immunological differences, which might be utilized as a supplemental marker for the differential diagnosis of the two hypersomnias.

Sleep alterations found in narcolepsy and idiopathic hypersomnia, such as disturbed or excessive sleep, could mediate the changes found in narcolepsy or idiopathic hypersomnia. In this case, the immune alterations reported here would be the consequence rather than the cause of the phenotype. Indeed, sleep restriction or deprivation has immunomodulatory effects, and alters serum IgG levels [18], [19]. Similarly, acute stress shows immune-enhancing and chronic stress provokes immune-suppression [20], [21]. A decline of sleep quality with chronic stresses induced by nocturnal sleep fragmentation and prolonged excessive daytime sleepiness might lead to the decrease in the serum total IgG levels in our narcolepsy patients.

A recent report found high titers of anti-streptolysin O antibody in narcolepsy patients within 3 years of disease onset [22]. Our findings of IgG alteration might underlie an increased susceptibility to specific infectious diseases. However we could not find any difference in disease duration between narcolepsy patients with and without abnormally low total IgG levels (27.3 years ±16.5, range: 9–48 years vs. 27.0±15.8, range: 1–52 years) and four narcolepsy patients within 3 years of disease onset showed no increased/decreased IgG levels. The observed IgG abnormality seemed to be disease independent and less likely the result of a putative infection.

In contrast to narcolepsy patients, idiopathic hypersomnia patients were likely to have increased slow-wave sleep [23]. Recent report showed that nocturnal sleep increases the percentage of IL-12 producing monocytes and concurrently decreases the percentage of IL-10 producing monocytes [24]. As one possibility, the increase in nocturnal sleep-associated predominance of type I cytokines might result in the augmentation of total IgG and the Th1 predominance in idiopathic hypersomnia patients. Several patients with symptoms similar to idiopathic hypersomnia were reported to have neural-specific anti-AQP4 antibody [25], [26], [27], and this organ-specific autoimmunity might be compatible with our findings of a Th1 involvement in idiopathic hypersomnia.

In narcolepsy patients, total IgG levels decreased, while the IgG3 subclass level stayed unchanged. IgG3 subclass appears first in the course of infection before other subclasses, probably reflecting the genomic position of gamma genes for the constant region in the immunoglobulin heavy chain locus. We speculate that the class switch from IgG3 to other IgG subclass in somatic rearrangement of the IGH gamma genes is dysregulated in narcolepsy. This dysregulation in the process of immunoglobulin maturation could also explain the decrease in IgG1 and IgG2 in narcolepsy with abnormally low total IgG levels. An immunoglobulin switch-like sequence was reported as a tight linkage marker for canine narcolepsy gene 1 [28]. It would be worthwhile to reanalyze human *mu*-switch region in detail, because the previous negative report of association using restriction fragment length polymorphism analysis might not examine the locus completely [29]. We observed elevated IgG4 ratio to total IgG in narcolepsy patients regardless of the total IgG levels. IgG4 abnormalities could be detected more efficiently when we used the proportion of IgG4. Two narcolepsy patients showed low IgG4 levels along with a marked reduction in IgG1 and IgG2. General immune-suppression may have occurred in these patients, similar to the case report of a narcolepsy patient who developed common variable immunodeficiency syndrome after the onset of narcolepsy [12]. However, these two patients took a typical clinical course of narcolepsy without severe immunological comorbid symptoms. The marked decrease in IgG observed in these two patients might be subclinical or somehow compensated.

Although no significant sex difference was observed in the mean total IgG levels in our study, which is consistent with results of a previous study [14], only male narcolepsy patients had abnormally low total IgG levels. We could not explain the reason for this gender difference. The mean total IgG level was marginally low in male HLA-positive controls compared with male HLA-negative controls (all: p = 0.016, limited to non-smokers: p = 0.032), while no difference was found in female controls according to HLA- DQB1*0602-positivity (p = 0.394). The combination of male sex and HLA-DQB1*0602 allele might bring forward the decrease of IgG level and sometimes form a subgroup of narcolepsy with abnormally low total IgG levels.

We could not match the possible confounding factors including age, sex, and *HLA* allele on the analysis of idiopathic hypersomnia patients with long sleep time, since the prevalence of idiopathic hypersomnia with long sleep time is low, estimated as only 2.0–4.1% of narcolepsy-cataplexy and female predominance were suggested [30], [31], [32]. Among these possible confounding factors, no sex differences were reported on total IgG levels [14]. As for the age, IgM and IgG levels were reported to be lower in children below 16 years compared with adults in general population [33]. However all our subjects were above 16 years except for two idiopathic hypersomnia patients, and these two patients showed normal total IgG levels. Thus the difference in age is not likely the cause of observed high levels of total IgG in idiopathic hypersomnia. As for the *HLA* allele contribution on IgG level, two idiopathic hypersomnia patients positive for the HLA-DQB1*0602 allele were examined separately, but did not show increased/decreased IgG levels compared to those negative for the *HLA* allele. Studies using larger number of samples positive for HLA-DQB1*0602 allele would be required to distinguish the *HLA* allele related effect and disease specific increase of IgG levels in idiopathic hypersomnia.

It would be necessary to evaluate the IgG levels in patients with narcolepsy without cataplexy, which was reported to have lower HLA association than narcolepsy-cataplexy and might have different IgG profiles. However we had inadequate number of narcolepsy patients without cataplexy after HLA-matching in this study. Also, it could be interesting if IgG levels in CSF samples be examined in future studies.

In conclusion, a significant decrease in the total IgG was observed in a subgroup of narcolepsy patients. We also found IgG subclass alterations in narcolepsy, suggesting IgG3- and IgG4-related abnormal IgG maturation in narcolepsy. The HLA-DQB1*0602 allele contributes to the decrease in the serum total IgG levels. The differences in the IgG profiles observed between narcolepsy and idiopathic hypersomnia indicate the immunological differences between these two conditions.

## Supporting Information

Figure S1 Influence of smoking for total IgG level (mg/ml). Subjects who smoke more than one cigarette per day are included in smoking group. Horizontal lines and squares indicate the mean ± SD. Numbers in graph indicate subject numbers in each group (female number is in parentheses). *P<0.05 vs non-smoking controls negative for HLA-DQB1*0602 (p values are as follows; smoking controls negative for HLA-DQB1*0602; p = 0.012, smoking controls positive for HLA-DQB1*0602; p = 0.005, smoking or non-smoking narcolepsy; p<0.0001). +P<0.005 vs IHS (p values are as follows; smoking controls negative for HLA; p<0.0005, smoking or non-smoking HLA-positive controls; p<0.005 and p<0.005, smoking or non-smoking narcolepsy; p<0.00001).
(0.03 MB PPT)
