# Supplementary material for: IgG Abnormality in Narcolepsy and Idiopathic Hypersomnia
Source: PLoS One. 2010 Mar 5;5(3):e9555. doi: 10.1371/journal.pone.0009555 (PMC2832686; doi:10.1371/journal.pone.0009555)
Supplement: Figure S1 — Influence of smoking for total IgG level (mg/ml). Subjects who smoke more than one cigarette per day are included in smoking group. Horizontal lines and squares indicate the mean ± SD. Numbers in graph indicate subject numbers in each group (female number is in parentheses). *P<0.05 vs non-smoking controls negative for HLA-DQB1*0602 (p values are as follows; smoking controls negative for HLA-DQB1*0602; p = 0.012, smoking controls positive for HLA-DQB1*0602; p = 0.005, smoking or non-smoking narcolepsy; p<0.0001). +P<0.005 vs IHS (p values are as follows; smoking controls negative for HLA; p<0.0005, smoking or non-smoking HLA-positive controls; p<0.005 and p<0.005, smoking or non-smoking narcolepsy; p<0.00001). (0.03 MB PPT) [file pone.0009555.s001.ppt]

## Slide 1
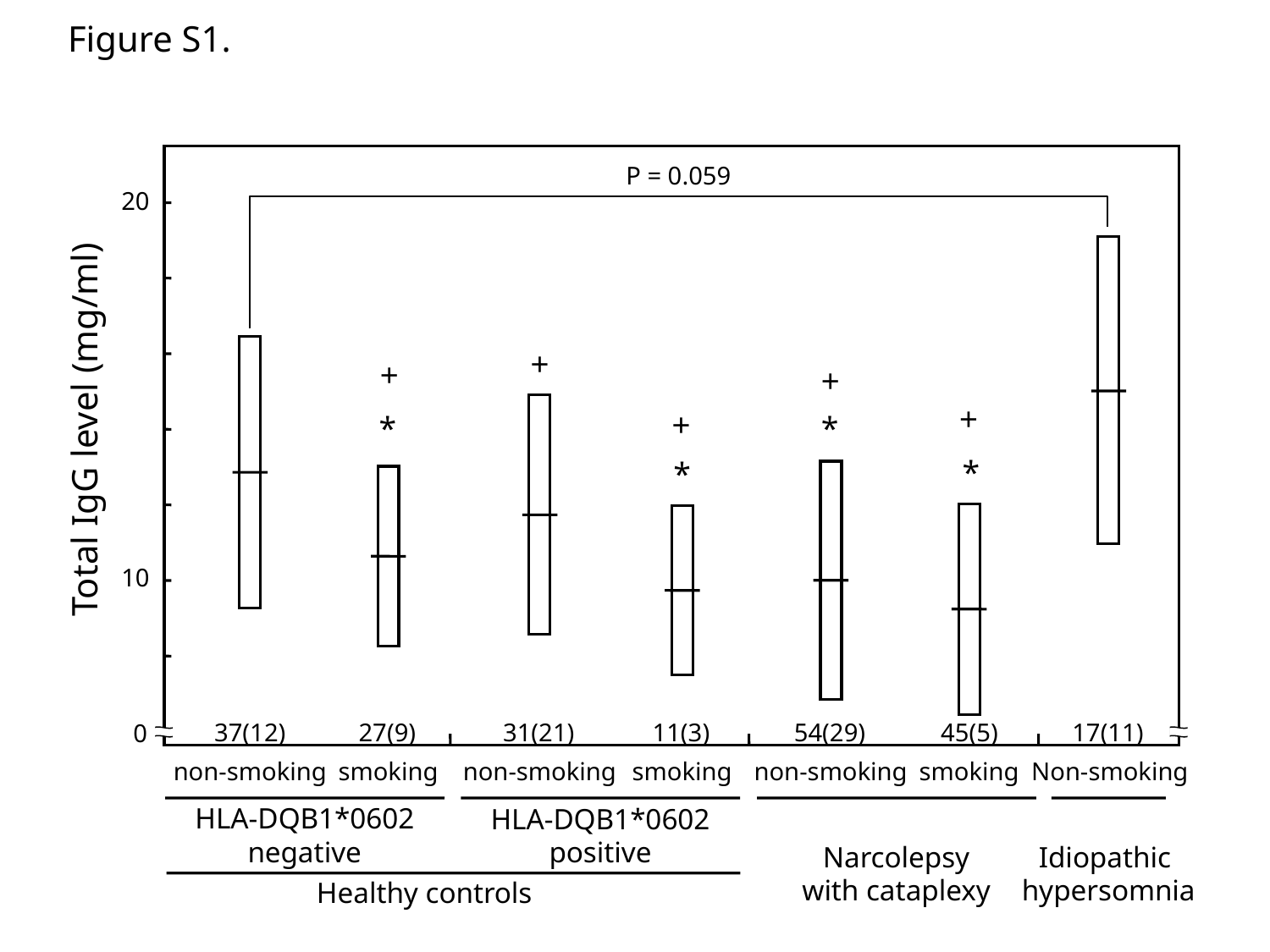

Figure S1.
P = 0.059
20
+
+
+
+
+
*
*
Total IgG level (mg/ml)
*
*
10
37(12)
27(9)
31(21)
11(3)
54(29)
45(5)
17(11)
0
non-smoking
smoking
non-smoking
smoking
non-smoking
smoking
Non-smoking
HLA-DQB1*0602
negative
HLA-DQB1*0602
positive
Narcolepsy
with cataplexy
Idiopathic
hypersomnia
Healthy controls
